# 5-HIAA as a Potential Biological Marker for Neurological and Psychiatric Disorders

**DOI:** 10.15171/apb.2019.044

**Published:** 2019-08-01

**Authors:** Hridya Jayamohananan, Maneesha Kalappurackal Manoj Kumar, Aneesh T P

**Affiliations:** Amrita School of Pharmacy, Amrita Vishwa Vidyapeetham, AIMS Health Sciences Campus, Kochi, India.

**Keywords:** Serotonin, Tryptophan, Raphe nuclei, 5-hydroxyindole acetic acid, Neurological functions

## Abstract

Neurological and psychiatric disorders occur in about 6 percent of the global population indicating a significant amount of people suffering from neurological disorder on a varying range in day to day life. On an extensive view, there is a critical requirement for the development of an alternative biomarker for these conditions. The thwart found in developing a biomarker is the difficulty in identifying a serum biomarker as these are mostly limited to the central nervous system (CNS). Serotonin being a neurotransmitter synthesized in the raphe nuclei of the brain could serve as an alternative biomarker. Here, the limitation is that it’s quickly metabolized by the mitochondrial enzyme MAO to 5-hydroxy indole acetic acid (5HIAA). This subsequent metabolite can be used for the analysis of serotonin levels in brain by analysing its concentration in the cerebrospinal fluid (CSF). Many theories suggest that the variations in serotonin level could lead to the development of many neurological and psychiatric disorders like Alzheimer’s disease (AD), schizophrenia, depression and so on. A decreased level is noticed in these patients but this could either be due to decreased production or increased reuptake of serotonin from the neuronal synapses. For instance, we know that a patient with depression shows a significant reduction in the levels of 5HIAA, due to the location of the raphe nuclei within regions of memory and cognition. Similarly, it does shows variation in AD and mild cognitive disorder. Evolving of 5HIAA as a biomarker, could be more delicate and enhanced strategy for monitoring these disorders.

## Introduction

Biomarkers, also known as biological markers are objective measures of biochemical or molecular parameters which are usually associated with the presence or sometimes the severity of a particular disease conditions or state. These biomarkers can be measurable mediums obtained by various techniques such as the physical examination, imaging or any laboratory assay. For neurological and psychiatric conditions, these agents are needed to be obtained directly from the cerebrospinal fluid (CSF) due to their close association with the brain and the central nervous system. These markers can show the potential biochemical changes taking place in the brain and evidencing its role as a potential biomarker.^1^

Different regular patterns for the biomarkers may emerge on regards to various diseases and its stages. A biomarker could serve as a risk-marker for the disease identification before or after its arrival, as seen in case of early detection Alzheimer’s disease (AD), the apolipoproteins (Beta amyloid, Aβ) isoforms. A biomarker could also serve as a disease- marker of the condition, that is, the variations would be noticed with the progress of the diseases and have also shown to fall to the normal levels on recovery. For example, the CA-125 known biomarker for ovarian cancer, increases on the onset of the neoplasia and fall on recovery. Disease marking biomarkers are preferred widely, as it portrays the underlying pathophysiological stages of the disease. On another scenario, biomarker could increase on the onset of a disease with the proportion of its severity indicating the damage caused by the disease, as in case of creatine kinase during myocardial infraction.

The aim of this study is to identify the serotonin metabolite 5-hydroxy indole acetic acid (5-HIAA) as a potential biomarker for the neurological and psychiatric disorders. The biomarkers such as serotonin can be helpful for early diagnosis along with severity and time course of the condition. For a better understanding, there required to shed some light on this synthesis and metabolites.

5-HIAA is known as a potential biomarker for the diagnosis and monitoring of carcinoid tumour of enterochromaffin cells of small intestine i.e., it a significant increase in the levels could be detected by urine sample analysis. On some other cases as autism spectrum disorders 5-HIAA levels are elevated. 5-HIAA being a serotonin metabolite produced in the brain from the tryptophan, which is among a few amino acids reaching the central nervous system would be able to give an insight on the activities taking place there.

Serotonin being a neurotransmitter (NT), is been widely distributed in our body and is found in various tissues such as the digestive tract, central nervous system and also found in platelets. Notably, it is found that only a small portion as about 1%-2% of the total serotonin is synthesized by the serotonergic neurons found in the brain, the rest 90% of serotonin is secreted by the enterochromaffin cells found in the gastrointestinal (GI) tract.^2^ On considering the serotonin levels of the blood, it’s been discovered that platelets are not responsible for synthesising serotonin by itself, but they just rather take it from the plasma, where it’s been released by the entero-chromaffin cells. The half-life of serotonin in plasma is the same as that of platelets (5-6 days). While in the central nervous system, it is mainly found in brain stem rather than the cortex region.^3^ During the neuro transmission, the release of serotonin from the pre-synaptic cleft activates a specific serotonergic receptor and then its reuptake is by the post-synaptic neurons.^4,5^

## The neuro-anatomical organization of the serotonergic cells in the brain

The neuronal cell bodies containing serotonin is found in a cluster of cells which are located along the mid-line region of the brain stem. According to Falck-Hillarp system of histo-fluorescence, a majority of the serotonergic soma is seen in the raphe nuclei. In a similar theory stated by Dahlstrom and Fuxe, the raphe nuclei of the brain comprise of about nine major serotonin-containing cell bodies which are primarily responsible for serotonin production in brain, apart from those found outside.^4,6^

## Synthesis of serotonin

**Figure 1:**
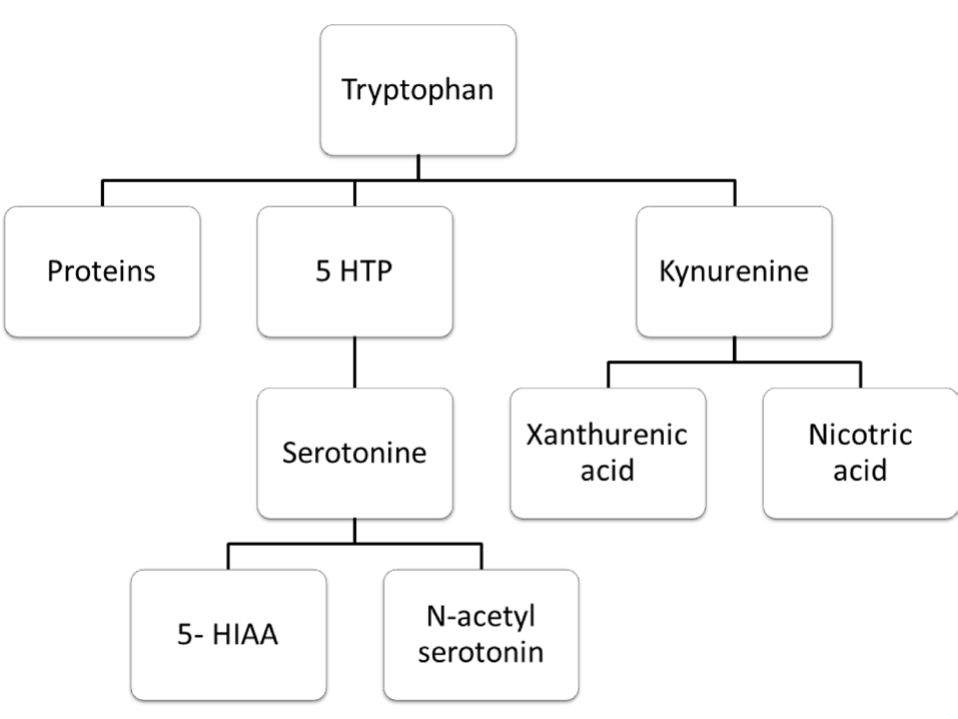
Biosynthesis of serotonin.

According to Pharmacorama, the 5 hydroxy tryptamine or serotonin is synthesized from an amino acid which is able to cross the blood brain barrier (BBB) as the L-tryptophan. Normally, the ingested quantity of tryptophan in an adult is about 0.5-1 g, whereas the advised daily allowance is around 200 mg, of which only a minor part is converted into serotonin as shown in Figure 1. The transformation of tryptophan into serotonin constitutes of two steps.^5,6^

(a) the hydroxylation of L-tryptophan which is catalysed by the enzyme tryptophan hydroxylase, (this enzyme is considered as the rate-limiting enzyme of this synthesis) to form 5- hydroxy tryptophan
(b) decarboxylation of the product of the latter step i.e., 5-hydroxyl tryptophan which is catalysed by amino acid decarboxylase with pyridoxal phosphate as the coenzyme.

**Figure 2:**
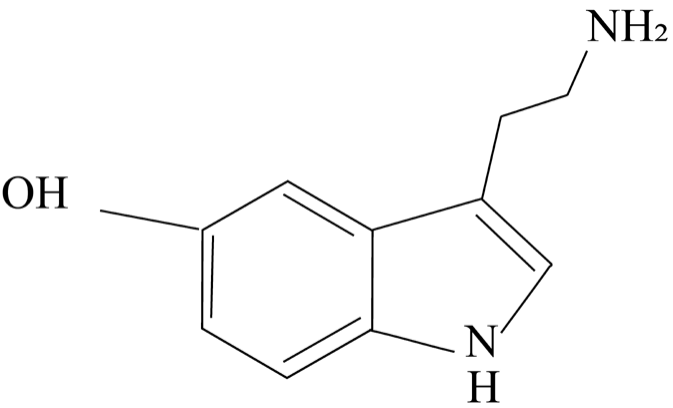
Chemical structure of serotonin.

**Figure 3:**
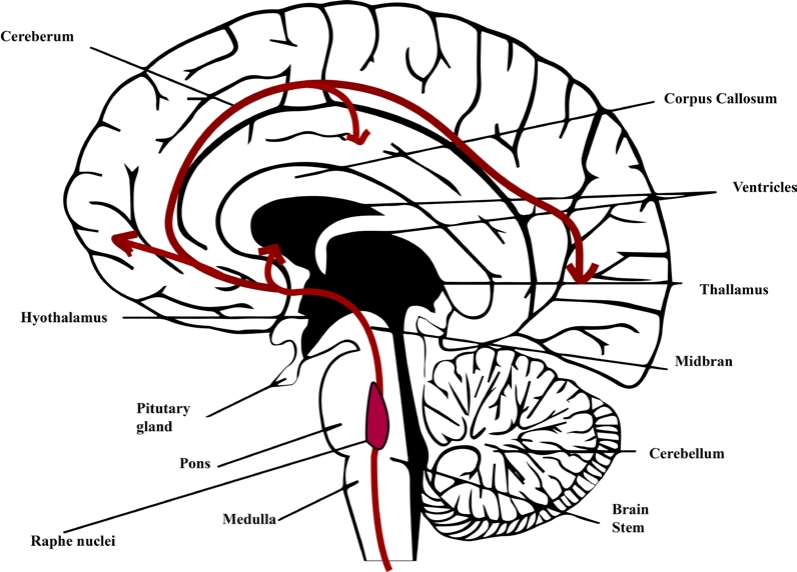
Pathway showing the production of serotonin in the brain.

As observed in Figure 2, in the chemical structure of serotonin, it is seen that a combination of a 5-hydroxyl group attached to the indole nucleus along with nitrogen of the primary amine is present which acts as a proton acceptor. Due to these reasons, 5HT is a hydrophilic substance and it cannot be passed through the lipophilic blood-brain barrier. However, its presence in central nervous system (CNS) has indicated that 5HT is synthesized in the brain. Certainly, not all braincells are responsible for synthesising serotonin. As mentioned, the raphe nuclei are responsible for the production of serotonin in the brain. The pathway of synthesis of serotonin is as illustrated in Figure 3. The major step involved in the synthesis of the 5HT is through the facilitated transport of the amino acid tryptophan, the synthesis of 5HT depends upon the amount of tryptophan which passes through the blood-brain barrier from the blood to the brain. The primary source of tryptophan is the protein-rich diet of which only the free plasma bounded ones can pass the BBB.^4^

## Serotonergic receptors

The serotonin receptor or 5 hydroxy tryptamine receptors, that are located both centrally and peripherally, comprising of G-protein coupled receptors and Ligand-gated ion channels with both excitatory and inhibitory functions.^5^ The NT serotonin is known to triggers these receptors. They are also known to regulate the release of other NTs including dopamine, acetylcholine, GABA, glutamate etc. It influences a various biological and neurological function such as aggression, appetite, cognition, anxiety, memory, learning^6^ nausea and even thermoregulation.^7^ The function with the corresponding responses of these receptors are enlisted in Table 1.

**Table 1:** 5 HT receptors and their response

**Family**	**Type**	**Mechanism**	**Function in CNS**	**Presence in CNS**	**Responses**
**5 HT**_1_	G_I_/G_0_ Proteins coupled	Decrease in cellular level of cAMP	Inhibitory	Yes	Addiction, aggression, anxiety, memory, mood, sleep, sociability
5 HT _1A_					
5 HT _1B_				Yes	Addiction, aggression, anxiety, memory, learning, mood
5 HT _1D_				Yes	Addiction
5 HT _1E_				Yes	-
5 HT _1F_				Yes	Migraine
**5 HT**_2_	G_q_/G_11_ Proteins coupled	Increase in cellular level of IP_3_ and DAG	Exicitary		
5 HT _2A_				Yes	Addiction, anxiety, cognition, imagination, memory, mood, sleep
5 HT _2B_				Yes	Anxiety, sleep
5 HT _2C_					Addiction, anxiety, mood
**5 HT**_3_	Ligand gate Na^+^ and K^+^ cation channels	Depolarizing plasma membrane	Exicitary	Yes	Addiction, anxiety, learning, memory
**5 HT**_4_	G_s _ Protein Coupled	Increase in cellular level of cAMP	Exicitary	Yes	Addiction, learning, mood
**5 HT**_5_	G_i_/G_0_ Proteins coupled	Decrease in cellular level of cAMP	Inhibitory		
5 HT _5A_				Yes	Sleep
5 HT _5B_				No	Pseudogene in humans
**5 HT**_6_	G_s _ Protein Coupled	Increase in cellular level of cAMP	Exicitary	Yes	Anxiety, cognition, memory, learning, mood
**5 HT**_7_	G_s _ Protein Coupled	Increase in cellular level of cAMP	Exicitary	Yes	Anxiety, memory, mood

## Metabolism

As indicated by Pharmacorama, the formed serotonin is metabolized chiefly by the mitochondrial membrane enzyme i.e., the monoamine oxidase (MAO), which are seen in two molecular types mainly the MAO-A and MAO-B.^8^ These varieties are noticed in both the peripheral and central tissue. The action of MAO is primarily responsible for the metabolism of serotonin and thereby forming 5 hydroxy indole acetaldehydes, which gets readily converted by the enzyme aldehyde dehydrogenase to the form the major metabolite, 5-hydroxyindoleacetic acid (5-HIAA). Thus, serving as a remarkable milestone, and providing a stable means to measure the amount of serotonin in our body.^9-11^

The CSF compartment which on being anatomically in contact with the brain, could niftily serve as a potential source for monitoring various crucial biomarkers that are known to be generating factors during the pathogenesis of CNS disorders.^12^ The commonly accepted approaches for analysing the CSF includes reversed phase high performance liquid chromatography (rpHPLC), microdialysis, positron emission tomography tracers etc. According to recent hypothesis, the NTs including serotonin and its subtypes as the 5 HIAA is known to increase in concentration with rise of ‘Stress’ or could show a decreased level in the alcoholic conditions.^13,14^ There are several theories linking with the variations of the serotonin level and development of new neurological disorders like schizophrenia and depression.^15^

## Depression

As per US Department of Health and Human Services, National Institutes of Health, National Institute of Mental Health,^16^ depression is one of the most common and serious forms of mood disorders; it can be defined as a low mood or aversion towards activity, personal thoughts, and behaviour. Depression is characterized by depressed mood, hopelessness, helplessness, guilty, difficulty in remembering details, difficulty in making up the decision, insomnia and even leads to attempt to suicide.^17,18^ According to NAMI,^19^ the National Alliance on Mental Illness, for some people it may be experienced once in their lifetime, but in most of the people, it recurs. In a study conducted on an estimate of 16 million American adults, almost 7% of the population experienced major depression episode the previous year. People of all age, races, ethnics, and socioeconomic background can experience depression. Women are more likely to be affected than men, around 70% women experiences depression; young adults are also the most prone age group for depression.^17-19^

Many researchers proposed the indoleamine hypothesis for depression. According to that, the vulnerability to depression and mania is due the decreased serotonergic activity in the brain. The reduction in the serotonergic activity can be either due to two reasons

(a) decrease in the serotonin release
(b) increase in the affinity of reuptake of serotonin from the neuronal synapsis.

The reduction in the serotonin level can be either because of decreased utilization of the protein-rich nourishment, or it could be due to degradation of tryptophan to kynurenine instead of converting to serotonin.

In addition to this, factor kynurenine is a retroactive agent which is also known to be responsible for the pathophysiological mechanism associated with depression. This exhaustion in serotonin improves the catabolism by chemicals such as tryptophan 2, 3-dioxygenase (TDO) in the liver^20,21^ and the indoleamine 2, 3-dioxygenase (IDO) in the lungs, placenta, blood, and brain.

The pro-inflammatory cytokines as interferon-c, transcriptionally induces the IDO enzyme that degrades the tryptophan and thus produces serotonin depletion. The reduction in the serotonin can be also due to the direct breakdown of tryptophan by IDO into N- formyl kynurenine then enters into a different metabolic pathway, which is known to complete with different fragment to that of the normal metabolic pathway with monoamine oxidase leading to a reduced conversion into the final end metabolite 5HIAA and causes a drive in the serotonin away from the degradation into 5HIAA and resulting in a reduction of 5HIAA.^22^

## Schizophrenia

According to Bressert,^23^ schizophrenia is the most common and complex form of functional psychosis, which is characterized by the combination of symptoms like delusion, hallucination, disorganized speech, a breakdown in the personal relationship between thoughts and reasoning along with emotional imbalances. According to the epidemiological studies done, the prevalence of the disease in the United States ranges from 0.6%-1.9%, and prevalence of the disease is found equal in both males and females.^24^ In the year of 1980, Tim Crow a British psychiatrist proposed a speculation, that was “The Crow’s hypothesis of schizophrenia”.^25,26^ As indicated in this, the theory of schizophrenia could be divided into two dimensions, so accordingly there are mainly of two symptoms, type 1 syndrome consist of positive symptoms of delusion, delirium, hallucination (auditory, olfactory, gustatory), thought disorder and bizarre behaviour, the symptoms that most of the individual does not experience, and type 2 consist of symptoms that are deficit of the normal emotional response, which include alogia (poverty of speech), asociality (no interest in sociality), avolition ( lack of motivation) and anhedonia (inability to express pleasure). In which these negative symptoms exhibited is thought to be due to the structural abnormalities of the brain including the cortical atrophy and ventricular damage.^25^

There are theories suggesting the role of serotonin in schizophrenia which have been proposed decades prior, which was with the perception with lysergic acid diethylamide^27,28^ (a peripheral 5HT antagonist), using this Gaddum, Shaw, Wooley (1954) suggested that schizophrenia might be due to the decrease in serotonin level in the brain. Certain other investigators have likewise proposed a hypothesis known as the “transmethylation hypothesis of schizophrenia”, which expresses that the regularly occurring biogenic amines like indolamine methylates into the shape of a methylated amine which leaves a daydreaming effector a hallucinating impact in schizophrenic patients.^29-31^

## Alzheimer’s disease

Alois Alzheimer, a German psychiatrist and neuropathology was credited with identifying the first case of “presenile dementia” which was later identified as Alzheimer’s disease. Alzheimer’s disease (AD) is a neurodegenerative disorder that affects the brain; this disease is most commonly seen in the elderly population.^32,33^ The disease is characterized by memory loss, disturbance in speech, disorientation further leading to eventual dementia.^34^

The basic pathophysiology features of AD are extracellular amyloid plague and presence of intracellular neurofibrillary tangles.^35^ According to the Alzheimer’s Association, the recognized pathophysiology was that there is presence of synaptic degeneration, hippocampal neuronal loss, and aneuploidy and seen in the affected brain, also several studies have demonstrated that altered cytokine level and associated dysregulation of kynurenine metabolism plays a role in the neurodegeneration, and the upregulation kynurenine are observed in a variety of neurological disorder. Among the neurological changes in AD, there occurs an elevation in IFN-γ, TNF-α, IL-1β, IL-2, and IL-8 along with this and decrease in the serotonin and increased kynurenine level. Similar changes are observed in the post mortem brain which also showed an increase of IL-6.^36-38^

Considering the recent development in the treatment methodologies there are many studies being carried out using the principle discussed above that is to make the cell more available to serotonin. According to the preclinical and clinical studies, the seven families of the serotonin receptor and serotonin play a major role in different aspects of cognitive dysfunction. Among the 7 families, the 5HT_6_ receptor has shown an enormous expectation to act as a drug target for the developing the cognition and therefore play an immense role in the treatment and the inhibition of these receptors leads to the improved cognitive function.^39,40^ In the recent day’s researches, it has been claimed that the 5-HT_6_ receptors play a major role in the Fyn signalling along with the amyloid proteins which serves as a main course for the development of AD.

It has always been known that several metabolites of tryptophan pathways involving either kynurenine or serotonin metabolism are the implication of neuroprotection or for regulation of brain Aβ levels or other functions as cognitive or synaptic maintenance. Thus, a several known metabolites of both kynurenine and serotonin metabolism pathways are known to suspect be involved in the neurodegenerative of AD. However, the imbalance between the pathways are regulated by a quite wider range of factors with kynurenine pathway and with a in parallel serotonin synthesis. 5–HIAA on being the dead-end inactive product of the serotonin metabolism was used here. As a result of this test it was found that the serotonin measurable metabolite 5-HIAA at a standard dose of 100 μM shows an increase in NEP activity with the change in time.^37^

Another study claims that serotonin receptor agonist when given in combinations with the antagonist can lead to an effective and a better therapeutic outcome in the patients with AD. According to a study by Morimoto et al, reported that the CSF levels of 5-HIAA, and 5- HT were reduced significantly in the patients with AD and thereby proofing that the decrease in the specific brain proteins as t-tau, p-tau and Aβ 1–42 along with the combination of 5-HIAA and HVA would lead to high diagnostic accuracy.^38^

## Mild cognitive impairment

According to the Alzheimer’s Association, mild cognitive impairment is defined as the decrease in the cognitive ability, which includes memory and learning, hence people with these tend to be more prone to conditions like Alzheimer’s and dementia. The long-term episode of memory and cognition is supported by the structural changes in the brain mainly found in the temporal lobe and memory system, which includes hippocampus, dentate gyrus and the cortical region of the extra hippocampal; these are the areas of the brain responsible decision making, also guided by the parietal cortex within the temporal lobe. The presence of serotonergic receptors is widely seen in these regions, and hence it has an influence of serotonin in learning and memory. The level of serotonin is maintained by dietary intake of amino acid tryptophan; hence, the decrease in the intake eventually decreases the availability of serotonin in the brain. Studies conducted showed that depletion of tryptophan consumed caused contextual memory in mice, loss of objective memory in the rat, declared memory in man.^39-42^

## Delirium

Delirium comes from the Latin term *de* meaning away from and *lira* meaning earth thrown in between two furrows. It is defined as a reversible transient cerebral dysfunction demonstrated as a wide range of neuropsychiatric indications. Here an individual’s performances are lower than their normal line on consciousness and the noted symptoms are waning and waxing. The state could be best described as an acute change in concentration, cognition and attention where awareness and vigilance are fluctuating.^43^ About 12 to 43 percent can be recognized and its estimated that about 80% of these patients and inadequately treated. It’s said that about 20% of the patients older than the age of 65 years are facing a complicated hospital stay due to delirium. Distractible attention, labile or fearful emotional feelings hallucinations etc are used to describe the condition. In clinical practise the clouding of consciousness or the occurrence of disorientations favour in differentiating between delirium and psychosis.^44^ Sleep is vital to keep up for the maintenance of the memory circuits, in the absence of sleep the brain could suffer progressive synaptic weakness. Cognitive impairment and mood swings are the earliest manifestations and could cause sleep deprivation leading to visual and other perceptible hallucinations. The regions as occipital, parietal and temporal lobe contribute to the visual hallucination which contributes to 30% of the total incidence.^45^

The exact pathophysiology of delirium is still unknown despite its large clinical impact. But researches have shown the effect of the NT serotonin on being an important excitatory NT has been able to through some light to this theory. The level of NT serotonin depends on its precursor amino acid tryptophan. It has been postulated that the decrease in the level of tryptophan leads reduction in the levels of serotonin and this finally chain to the development of delirium. There is a linkage between many NT which on the reduction in the levels of amino acid could play a role in the incidence of delirium. As for example, the amino acid tryptophan and phenylalanine compete with each other for the transport across the BBB, so a change in these ratios of these amino acid leads to the decrease or increase in the levels of serotonin, which could probably cause delirium.^46^

The imbalance of serotonergic system is one of the potential mechanisms for delirium. An elevation in the values could be noticed caused but the selective serotonin reuptake inhibitors acute withdrawal effects. In a study conducted by Robinson et al, the no of patients with postoperative surgery who had developed delirium was having a much lower levels amino acid tryptophan to the number of postoperative surgery patients who have not developed delirium.^47^ According to a study by Banki and Vojnik, it found that the CSF levels of 5- HIAA along with the serum serotonin levels on the clinical studies conducted on patients suffering from delirium tremors and other schizophrenic patients with acute clozapine induced delirium on simultaneous studies showed a significant raise.^48^ The readings where returned to normal range on recovery. Lesions found in regions as the basal ganglia, thalamus, temporal or parietal where found to contribute to the delirium cases.

## Analysis of the biological sample

An accurate measurement of serotonin (5HT) and its metabolites as 5-HIAA have always served as a field of interest for reviewing in the internal medicine, psychiatry and its other pharmacological effects.^49^ The measurements of 5-HIAA in the urine sample serve as a biomarker for analysing or diagnosing carcinoid carcinoma and CSF analysis could be useful for predicting different psychological and neurological conditions.

A potential approach in the serotonergic studies of 5-HIAA measures are found uniform to the subjects of earlier studies shows a clear-cut view on the relevance of serotonin on these disorders. The serotonergic dysfunction is closely associated with aggressive and impulsive behavioural aspects.^50,51^ For the CSF 5-HIAA analysis, includes the initial procedure for collection and processing of the sample obtained from polypropylene test tubes (2000 g) which are meant to avoid the absorbance of different proteins and peptides found mainly at the test tube walls.^52^

For the analysis, the sample as the blood, urine, CSF collection protocols must be followed. The obtained sample must be sent for analysis within 30 min of the draw, and the remaining Aliquots could be stored frozen at a temperature of -80°C.^52,53^

There have been many analytical techniques used for monoamine metabolites as serotonin and its metabolites. These could include the capillary get-electrophoresis, High profile liquid chromatography hyphenated by mass spectrometry (HPLC/MS), Gas chromatography hyphenated by a mass spectrometry (GC/MS) or HPLC with electrochemical (EC) detection. The current method used for CSF analysis and its metabolites mostly rely on the rpHPLC coupled with an EC detector. These systems are extremely sensitive, selective and stable device used generally under isocratic conditions with a reversed phase column with or without an ion pairing incorporated with the mobile phase.^54^

The variations in the pH and other organic modifying factors as methanol or acetonitrile concentration could affect the retention time of the metabolites, which could be used for the optimum separation of the constituents. The accuracy in the measurements of CSF 5-HIAA, mainly depends on the separation of other constituents form the sample which could be resolved by partial purification but when there is a need for measuring of large number of these samples would serve as challenging. Thus, an alternative could be a multielectrode coulometric electrochemical cells connected in series with a gradient elution capability, working on the current/voltage characteristics of the sample.

## Conclusion

As already discussed, serotonin does play a major role in maintaining the normal psychosis; hence, the variation of this NT produces a disturbance in the normal psychotic function. The level of serotonin in blood varies from that of the brain, so to analyse the amount of serotonin we use a sample of CSF. Since serotonin undergoes rapid degradation to 5-HIAA, this is used as the parameter to evaluate the amount of serotonin in the brain. According to various studies conducted, all disease conditions discussed above shows a clear reduction in the levels of 5-HIAA. Now it is evident that serotonin does serve as a potential biomarker for many of the neurological and psychiatric disorders, thus giving a wider scope of future studies in developing an alternative biomarker.

## Ethical Issues

Not applicable.

## Conflict of Interest

Authors declare no conflict of interest in this study.
